# A global approach to the management of EMR (Electronic Medical Records) of patients with HIV/AIDS in Sub-Saharan Africa: the experience of DREAM Software

**DOI:** 10.1186/1472-6947-9-42

**Published:** 2009-09-11

**Authors:** Andrea Nucita, Giuseppe M Bernava, Michelangelo Bartolo, Fabio Di Pane Masi, Pietro Giglio, Marco Peroni, Giovanni Pizzimenti, Leonardo Palombi

**Affiliations:** 1 Dipartimento di Fisica, Sezione Informatica, Università degli studi di Messina, Messina, Italy; 2 Telemedicina, Azienda Osp. S. Giovanni Addolorata, Roma, Italy; 3 Comunità di Sant'Egidio, Roma, Italy; 4 Dipartimento di Sanità Pubblica e Biologia Cellulare, Università di Roma Tor Vergata, Roma, Italy

## Abstract

**Background:**

The DREAM Project operates within the framework of the national health systems of several sub-Saharan African countries and aims to introduce the essential components of an integrated strategy for the prevention and treatment of HIV/AIDS. The project is intended to serve as a model for a wide-ranging scale-up in the response to the epidemic. This paper aims to show DREAM's challenges and the solutions adopted. One of the solutions is the efficient management of the clinical data regarding the treatment of the patients and epidemiological analyses.

**Methods:**

Specific software for the management of the patients' EMR has been created within the DREAM programme in order to deal with the challenges deriving from the context in which DREAM operates. Setting up a computer infrastructure in health centres, providing a power supply, as well as managing the data and the project resources efficiently and reliably, are some of the questions that have been analysed in this study.

**Results:**

Over the years this software has proved that it is able to respond to the need for efficient management of the clinical data and organization of the health centres. Today it is used in 10 countries in sub-Saharan Africa by thousands of professionals and by now it has reached its fourth version. The medical files of over 73,000 assisted patients are managed by this software and the data collected with it have become essential for the epidemiological research that is carried out to improve the effectiveness of the therapy.

**Conclusion:**

Sub-Saharan Africa is the region hardest hit by HIV and AIDS in the world. However, the resources and responses adopted so far, to confront the epidemic, have at times been rather minimalist. The DREAM project has faced the battle against the epidemic by equipping itself with qualitative standards comparable to Western ones. The experience of DREAM has revealed that it is indeed possible to guarantee levels of excellence in developing countries, also in the sphere of ICT (Information and Communication Technology), thus making the intervention even more effective and contributing to bridging the digital divide.

## Background - The DREAM Programme

Drug Resources Enhancement against AIDS and Malnutrition (DREAM) [1] was created by the Community of Sant'Egidio to fight AIDS in sub-Saharan Africa. The project takes a holistic approach, combining Highly Active Anti-Retroviral Therapy (HAART) with the treatment of malnutrition, tuberculosis, malaria and sexually transmitted diseases. It also strongly emphasizes health education at all levels. DREAM aims to achieve its goals in line with the gold standard for HIV treatment and care.

DREAM was launched in Mozambique in March 2002, following two years of groundwork. However, the idea for the project was born in 1998 when the Community of Sant'Egidio- a Christian movement founded in Rome in the late 1960s that has a strong base in Africa - decided to fight the devastating impact of HIV/AIDS. DREAM has now spread to 10 African countries: Mozambique, Malawi, Tanzania, Kenya, the Republic of Guinea, Guinea Bissau, Cameroon, Congo RDC, Angola and Nigeria.

### What DREAM is about

This pandemic has characteristics that make it unique in its kind and which can be summed up as follows:

• The HIV/AIDS infection is mainly concentrated in countries with limited resources, and in particular, in sub-Saharan Africa. It has become the first cause of death here and the new infections per year still outnumber the deaths. In fact, according to the WHO statistics, there are 33 million infected people in the world, around 60% of whom live in sub-Saharan Africa. Every year it is estimated that around 2 million people die from HIV/AIDS, and over 70% of them in sub-Saharan Africa. [2].

• No vaccines are available yet, neither preventive nor therapeutic. None the less great progress has been made in the field of antiretroviral drugs, which have been administered in a triple combination since 1996 and have reduced the death rate in the West by 90% [3].

• However, the antiretroviral drugs are not able to eradicate the virus: therefore the patients' health depends on them taking the drugs for their whole life. One can thus understand that the richness and complexity of the clinical records generated and of the history of the patients is unequalled by any other pathology.

• Moreover, both the infection and the therapy need to be carefully monitored with a sophisticated diagnostic system organised on four levels: progression of the disease and the patient's clinical condition; his immune status (mainly expressed in terms of his CD4 cell count); the "quality" of the viral infection (viral load and resistance mutations); surveillance for any adverse events and toxicity related to the pharmacological treatment.

• Another critical point is that the whole system has to be integrated into the health systems of countries with limited resources and has to take into consideration other widespread conditions in these countries, like for example, malnutrition, the low level of access to health services and the poor level of health education [3].

• One more point is that the poor knowledge about the disease and above all the need to consolidate solutions for several aspects of the public health system (like for example, the prevention of mother-to-child transmission, the knowledge of the best time to start therapy, the control and prevention of co-infections and of opportunistic diseases) mean that the African programmes have to be able to carry out research, in particular operational research. This then means that the data collected have to be available for drawing up reports, but also for data mining, cost/benefit evaluations, and epidemiological analyses in general.

• Finally DREAM had to deal with another key problem in sub-Saharan Africa, which is the dramatic shortage of qualified health staff, as reported recently by the WHO. In other terms both the clinical centres and the laboratories had to combine adequate apprenticeships with the theoretical training of new biologists, doctors, laboratory technicians and obviously computer experts [2,3].

• Considering the above, the DREAM Project aims to introduce the essential components of an integrated strategy for the prevention and treatment of HIV/AIDS within the framework of national health systems. The project is intended to serve as a model for the wide-ranging scale-up of the response to the epidemic. Its main objective is achieved through the establishment of services providing diagnosis and comprehensive treatment.

The prevention of HIV transmission in the general population and of mother-to-child transmission through Community Care and Home Care services (CCHC) and Mother and Child Prevention and Care (MCPC), respectively, are additional key components of the programme. The Community Care and Home Care services look after the rest of the family, that is the male partners in the first place, and also the children. Mother and Child Prevention and Care is DREAM's approach for achieving results in caring for the mothers and preventing mother-to-child transmission. This link is crucial to ensure the survival of women and good adherence to the treatment programme.

Since adherence to ARVs and treatment follow-up are essential for the effective use of HAART in large-scale public health settings, DREAM provides the treatment package free of charge to all patients. This is a crucial element; for many patients, even the cost of transport may prevent them from adhering to treatment. By eliminating the cost of treatment, high adherence rates have been achieved [4,5].

### Typology of patients, modality of intervention (MCPC, CCHC)

DREAM realized early on that it was not feasible to offer treatment indiscriminately to everyone with HIV. In order to use limited resources most efficiently [6,7], the project gives preference to carefully selected groups of patients:

• **Pregnant women**, to prevent mother-to-child transmission and ensure the mothers' survival.

• **Skilled workers**, especially in the health sector, such as doctors, nurses, laboratory technicians and other personnel. Today more than 170 employees in the health sector are receiving treatment.

• **Employees of certain businesses**, because DREAM favours collaboration with businesses that offer antiretroviral treatment to their employees and their families in order to avoid the loss of skilled personnel. This decision aims to preserve the economic and productive fabric of the country. The collaboration generally consists of financial co-sponsoring of treatment for the employees of these companies. In some cases, doctors employed by the firms are trained in providing HIV treatment. This enables the doctors to manage free-of-charge clinics - in premises made available by the same companies - where antiretroviral treatment is provided. The doctors are further supported in this initiative with, for example, free access to laboratory testing.

• **Patients living within the catchment area of the centres**, all the patients who live near health centres have access to the treatment, regardless of the above mentioned criteria but respecting the limits of each centre's capacity.

### Basic concepts and programme expansion

DREAM has chosen to invest in intensive short-term personnel training, followed by more prolonged in-service training with the support of expert personnel at the workplace.

**Figure 1:**
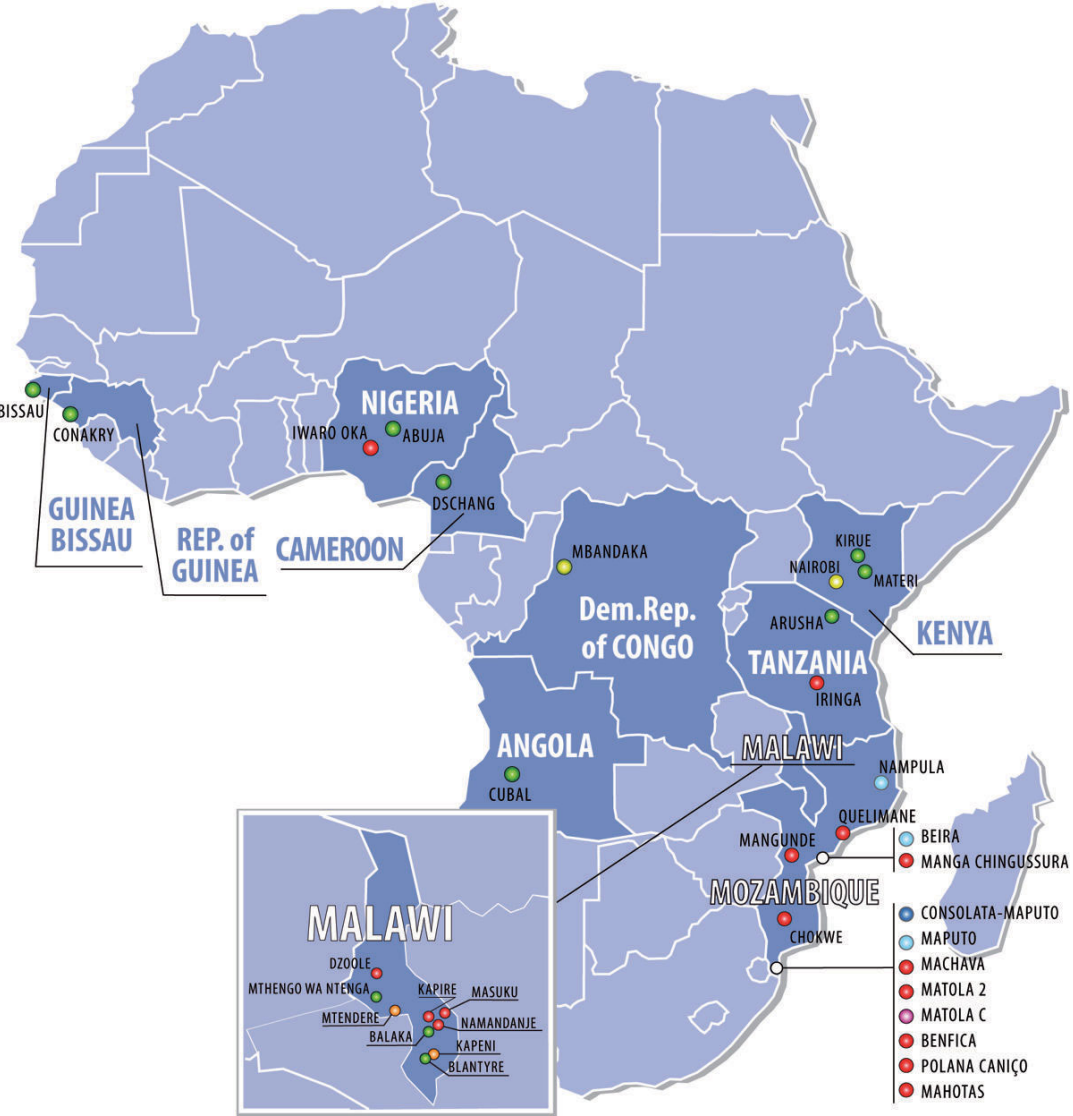
**Expansion of DREAM Centres**.

Community intervention models have also been developed, such as outpatient care, community and home-based care. These do not require extensive resources and can be set up quickly. As a backup, a small number of high technology centres capable of supporting large areas (e.g. molecular biology laboratories) have been set up too. A third step has been to invest heavily in the provision of access to treatment and clinical and laboratory monitoring for patients in remote locations. Centres within a 150 km radius of cities have been equipped so that they can offer the same qualitative level of treatment and monitoring as those closer to the cities. Apart from supplying equipment, the transport of blood samples and periodic supervision by DREAM staff are also organized. The fourth step consists of the effective use of computer resources to guarantee both the best possible organization of the work and even long-distance monitoring of various aspects of the programme. An efficient computer system also makes it possible to provide additional support to clinical and diagnostic operations through the use of Internet technologies. This may include second opinions from specialized European centres, telemedicine, telediagnostics and long distance training. The elements of this comprehensive model may be duplicated to treat conditions other than AIDS, such as tuberculosis. Hence, the model may potentially serve as a blueprint for the development of a broader response by health systems to HIV in resource-limited settings. The scalability of the programme has given it the possibility to expand rapidly: as already mentioned, the programme has now spread to 10 countries of sub-Saharan Africa, with 31 centres and 18 molecular biology laboratories that are already operational (Figure 1). It has been calculated that since 2002, the year that the programme started, over 73,000 patients have received assistance in the DREAM centres. Table 1 shows some indicators regarding the clinical examinations carried out, samples processed, analyses performed, etc. and this gives an idea of the extent of the activities related to this population (Table 1).

**Table 1:** The DREAM figures from 2002 to today

Type of assistance	Quantity
Patients under treatment	73,000
Medical visits carried out	450,000
Viral loads carried out	180,000
CD4 carried out	440,000

More generally, one has to bear in mind that with its health education and nutritional and social support, DREAM has met and supported an even wider population, including the patients' families and their original communities (villages or districts), which amounts to around one million people. The present and future contacts with this population are obviously extremely numerous and require a network of mobile staff who can guarantee and protect accessibility to the various services, adherence to the therapies and quick reporting of health emergencies.

This study presents the effort made within the programme to create the software to manage the clinical data and the computer infrastructures necessary for its use. The rest of the paper is organised as follows: the Methods section discusses some basic matters regarding the creation of the DREAM software; the Results and Discussion section presents the out come of the use of the software; finally the Conclusions offer some considerations matured in this paper.

## Methods

**Figure 2:**
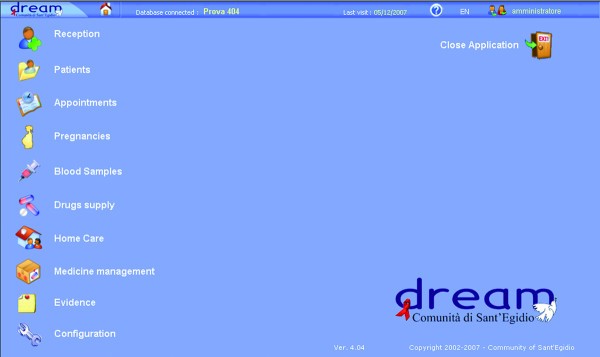
**A Screenshot of the DREAM Software's Main Form**.

The rapid expansion of the programme, and therefore the need to deal with a vast amount of clinical data, has led to the creation of specific software in order to manage the patients' medical files and their diagnostic data, called DREAM Software (Figure 2 shows the Home page of the software). Below we show the challenges involved in designing this software, how it has become an essential instrument for treatment and for epidemiological analyses and how it can, like DREAM as a whole, become an instrument that can be used in similar contexts.

### Basic concepts

DREAM Software was born of the need to computerise the management of the clinical files of DREAM patients.

This need envelops three main objectives:

i. Optimising access of the patients to the clinic so as to guarantee the highest possible number of daily visits;

ii. Having at one's disposal a database with information on the clinical history of individual patients and on the overall running of the DREAM centre, which is useful for refining therapies and for the sound running of the centres;

iii. Providing researchers with a useful database, by making use of the experience accumulated over the years.

The first objective of DREAM, and consequently for DREAMS (DREAM Software), is to guarantee a standard of quality comparable with that of developed countries. In this sense, the genesis of the software is strictly related to that of DREAM.

It has been recognised [8,9] that developing computer projects for application in the health services sphere is a very complex activity, which must necessarily take into account the perspective of the end user, who is involved in the iterative process for the development of software tools [10,11]. DREAM Software was created and developed to respond to the needs which emerged in the field and has been elaborated with experience. Hence the end users were extensively involved, and they gave a fundamental contribution to the creation of the tool which they themselves would be using. In fact, during the development of the project, a selected group of users (including doctors, nurses, biologists and technicians) had access to a website specially developed to collect comments and requests regarding the software. This way the team of developers were able to receive suggestions and indications from users in various countries, coordinating the modifications and improvements to the software. The users classified all the comments they made by giving them a priority value in order to direct the software designers towards the most urgent matters.

**Figure 3:**
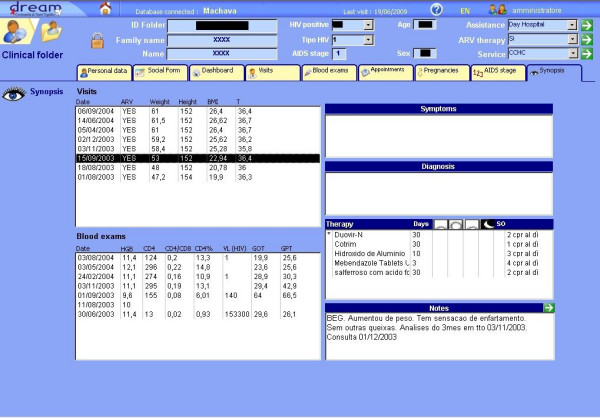
**A Screenshot of the DREAM Software's Synopsis Form**.

For example, one important aspect of the treatment of AIDS, as already mentioned, is the fact that it will last the whole of the patient's life. From the beginning of the therapy, the patient may have dozens of medical examinations and tests, which it is important to evaluate as a whole, in order to have an overall vision of the treatment of the patient. This is why the software includes a form, called Synopsis, in which the doctor has a complete and synoptic view of the various events that have occurred over the whole period during which the patient has been in treatment. This function was not present in the first version of the software and has been added thanks to the indications of the doctors who use it (Figure 3).

Moreover, the expansion of DREAM to several countries (with different languages) introduced a further level of complexity, that of sharing data [12,13]. In this sense, DREAM Software was also born of the need to introduce uniformity in the gathering of data by different centres in different countries, for the monitoring of centres and for the refining of therapies. Often, in fact, integrating data coming from heterogeneous sources is a problem which may require a somewhat complex solution [8,14]. The need to collect and share data for subsequent analysis required a solution to the communication problems often encountered in African countries.

The typical architecture of DREAM centres is illustrated below and then the solutions adopted to solve the problems of communication and information sharing is presented. The question of data management is then dealt with and this also plays a very important role in the treatment of patient and in epidemiological research.

### Architecture of the centres

This section briefly presents the typical architecture of a DREAM centre.

**Figure 4:**
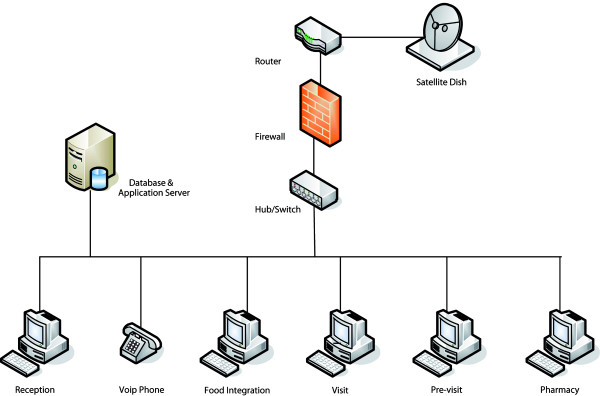
**Architecture of a DREAM Centre**.

The DREAM database and Software are on the computer server in every DREAM centre. For security reasons, access to the database can take place only indirectly through the DREAM Software: the users (the coordinator, doctors, nurses and operators) do not have the privilege of direct access to the database. This precaution is to prevent a malicious (or inexpert) user from damaging the database or from deliberately spreading sensitive information. Moreover, sharing the same software on the server simplifies the configuration of the computers used. The architecture of a DREAM centre is illustrated in Figure 4.

As may be seen, every service within the DREAM centres (the examination room, the pharmacy and so on) has a computer, which is linked to the server and to the rest of the computers through a LAN, giving individual operators the possibility of following the patient for his/her entire course of treatment in the centre, with the most updated data always at hand, without having to refer to files on paper.

Communication between the various centres is guaranteed by an Internet connection, and through satellite in those places where it has proved impossible to get connected through an already existing cable network. Communication has turned out to be of considerable importance for two fundamental reasons, namely the centralised compilation of data and communication/consultation with specialised personnel. Through Internet communication (instant messaging or VoIP), every user can communicate with the personnel of other African or European centres. This has made the extensive use of teleconsultation possible (with no additional cost save that of the Internet connection), and this is very useful when the doctor feels the need to interact with colleagues about more complex clinical cases.

### Difficulties in the setting and proposed solutions

When situated in a complex environment like the African reality, a structure of this kind is naturally subject to different kinds of problems: power surges, disruptions in network connections, both LAN and Internet and low bandwidth. Years of experience in this field have led to the identification of simple solutions to these problems. Clearly, a distinction needs to be drawn between the big centres, found in large cities where the problem is usually just of an economic/financial nature, and rural centres where the challenges are of a technical nature. The most pressing problem was undoubtedly that of power current; we can list three types of solutions of increasing complexity: i) using a UPS, ii) using a system of (rechargeable) batteries and of inverters, iii) using solar panels and batteries. The first method is obviously effective only in those cases where the power supply is present but erratic, and the dimensions of the UPS may be measured on the basis of the estimated quality of the connection. The second system is used in centres which are open only on some days, and it is possible to recharge the batteries in backup/support centres. The last is the latest method and allows for the continual use of our centres.

The problem of the quality of the LAN is fundamentally linked to the quality of the planning and implementation of the installation process. By consulting engineers, it has been possible to equip local technicians with the necessary know-how to create installations of a quality comparable to Western structures.

As for Internet connections, especially regarding bandwidths that are not always adequate, the matter is tackled in different ways. Careful enquiries made to local Internet service providers (ISP) have allowed many centres to have good quality Internet access at an accessible price. In cases where this was not an option, the problem has been resolved by using satellite connections, usually installed in our laboratories. For all the rural centres that are not connected to the Internet, it has been possible to transfer requests for tests via flash disk from the centre to the laboratory. The same applies to the transfer of backup of the centres. Once the backup reaches the laboratory, the software makes it possible to send all the backup of the satellite centres to a centralised server. Another important point: the size of files sent is reduced, to make them as robust as possible, so as not to be vulnerable to possible loss of data. An incremental backup system with especially strong redundancy was designed in order to achieve this.

Testifying to the quality of the networks (electrical as well as computer) of DREAM centres, VoIP communication systems have been introduced (at times supplied by the operators), which have allowed for the reduction in intercontinental teleconsultation costs.

### Data management

As already mentioned, data management is an extremely important aspect. Not only because by computerising the medical files the work in the centres is carried out more efficiently. In fact the data collected make up a real mine of information that is useful for operational research, in order to make not only the work of the individual health centres more efficient, but also DREAM as a whole. This means that there has to be a reliable database and that the data collected in the various countries where DREAM is active have to be homogenous.

A large part of the work on DREAM Software has been dedicated to the design of a relational database for the management of data contained in clinical files. The database consists of 42 tables, which contain data relative to the topics presented in Table 2. This is an important resource both for the management of the clinical data of patients, as well as for the possibility it gives to analyse information found therein, from a clinical and organisational point of view.

**Table 2:** Typology of data represented in the database

Topic	Description
*Statistics*	Information relating to statistical and social data of patients
*Examinations*	Data about examinations of patients with relative clinical parameters
*Tests*	Data about tests
*Symptoms and diagnosis*	Codification of symptoms and of diagnosis, dictionary of data ICD X
*Drugs*	Codification of drugs, prescriptions and delivery of drugs, dictionary of data (ATC)
*Home care*	Data about home visits to patients who enjoy this service
*Software users*	Data managing access to the software
*Log errors and warnings*	Codification of messages of log errors or warnings used in the software.

As has already been acknowledged [15-17], creating a dictionary of terms used is of crucial importance for the management of the database. In particular, a need was seen for encoding the most important information - pathologies and drugs although not exclusively - as this has the capacity of making such information usable in an epidemiological context while maintaining a friendly approach towards clinical users. ICD X and ATC codifications in particular were used. The transcoding dictionaries compile 2,700 items for pathologies in the following languages: English, French, Portuguese and Italian. This feature is all the more useful in the case of DREAM, where the presence of many centres spread across different countries makes the homogenisation of terminology necessary. In the DREAM Software, pre-codified data is consequently used for the specification of symptoms, of diagnoses and of drugs, to avoid the possibility of inserting free text which would lead to non-homogeneity of data.

The registration of test results coming from laboratories (which have specific software for the administration of tests) has been automated too, to prevent mistakes in copying values [18]. In fact, it has been noted that the quality of data (accuracy and completeness) is fundamental, among other things, to enable the integration of a support tool with decisions in the system [19]. We thus chose to use an established terminology, in order to create structured records that could be used towards this end. On the other hand, the input of the patients in supplying information about themselves has been recognised as very useful in several cases [20]. Consequently, the Software provides the option to add notes in fields for free text, apart from the codified data to be filled for each patient, so that news (possibly supplied by the patients themselves), which cannot be committed to memory in any other way, may be recorded there.

It has been noted [9] that the preservation of data relating to electronic health records (EHR) is a critical issue, even if at times the staff may adopt an attitude of mistrust with regard to computerized systems for patient management [21]. The security of data is consequently a fundamental aspect of the programme, bearing in mind that frequently data pertaining to DREAM patients on treatment is the only clinical data which exists about them. Hence, the database is equipped with a sophisticated backup and recovery system which guarantees the reliability of the system and the persistence of data.

Further, since the system handles sensitive, private information about the patients on treatment, we have sought to make the data secure on various levels, preventing its malicious diffusion or loss due to technical malfunctions. Despite the fact that many African countries have no specific regulations governing privacy, the data are dealt with according to the European regulations on privacy and electronic communications.

As already mentioned, a fundamental aspect of DREAM Software is that it links tools for epidemiological investigation to the automatic management of the patients' clinical files. A database containing the clinical data of patients on treatment provides the opportunity to use these data to conduct large-scale epidemiological investigations. This required the creation of a centralized database, in which to merge the data of the various databases of the DREAM centres. In order to keep this database updated, an additional backup procedure has been installed and it periodically (according to the settings) sends new data to the central database. A copy of this centralised database is then used for the analysis of data, through the use of specific tools.

Only the director and the scientific managers' medical staff have access to the central database, managed in the DREAM project's data processing centre in Rome. This way the data collected are used in an anonymous and assembled way for epidemiological research and for the evaluation of the therapy in the various countries' centres.

### Some of the software's features

Some instruments have been implemented in the software to make the staff's work quicker and more accurate.

This section intends to only describe some of the functions of the software but they are extremely important for its effectiveness. In fact every function has a crucial role for the success of the therapy and for the excellent management of the resources (human and economic).

- *Management of the appointments*. This function is very important because it makes it possible to rationalise the centres' work. It is therefore considered an organisational aspect.

- *Management of the drug storeroom and drug handover*. This part of the software is of paramount importance, since precision in administrating the therapy and in taking the drugs is fundamental for the success of the therapy. The drugs are also a very important resource from an economic point of view.

- *Managment of the languages*. Great attention has been paid to the creation of a multilingual environment, because of the possible presence of international staff collaborating in the same health centre.

These functions, which have been developed to respond to the requirements of the DREAM health centres, are briefly explained below.

*The appointments *with the patients are managed by the software, in order to help with coordinating the health centre, which often look after thousands of patients. There are also some automatic warnings to highlight certain situations regarding patients who need particular care. This way one has not only a complete view of every patient's situation, but it is also possible to monitor how the centre is doing as a whole.

*Managing the drug *storeroom, the prescriptions and the deliveries is all dealt with by the software. This makes it possible to rationalise one of the project's very important and expensive resources, the drugs, particularly the antiretroviral drugs. The efficient management of the drugs has turned out to be a fundamental aspect of a project like DREAM, for at least two reasons: (i) it improves adherence to the therapy by monitoring the patients' use of the medicines; (ii) it helps avoid any waste in using the drugs. In particular the first aspect is crucial, given the complexity of the AIDS treatment because of the difficulty in substituting the therapy in the case of resistance to the drugs.

**Figure 5:**
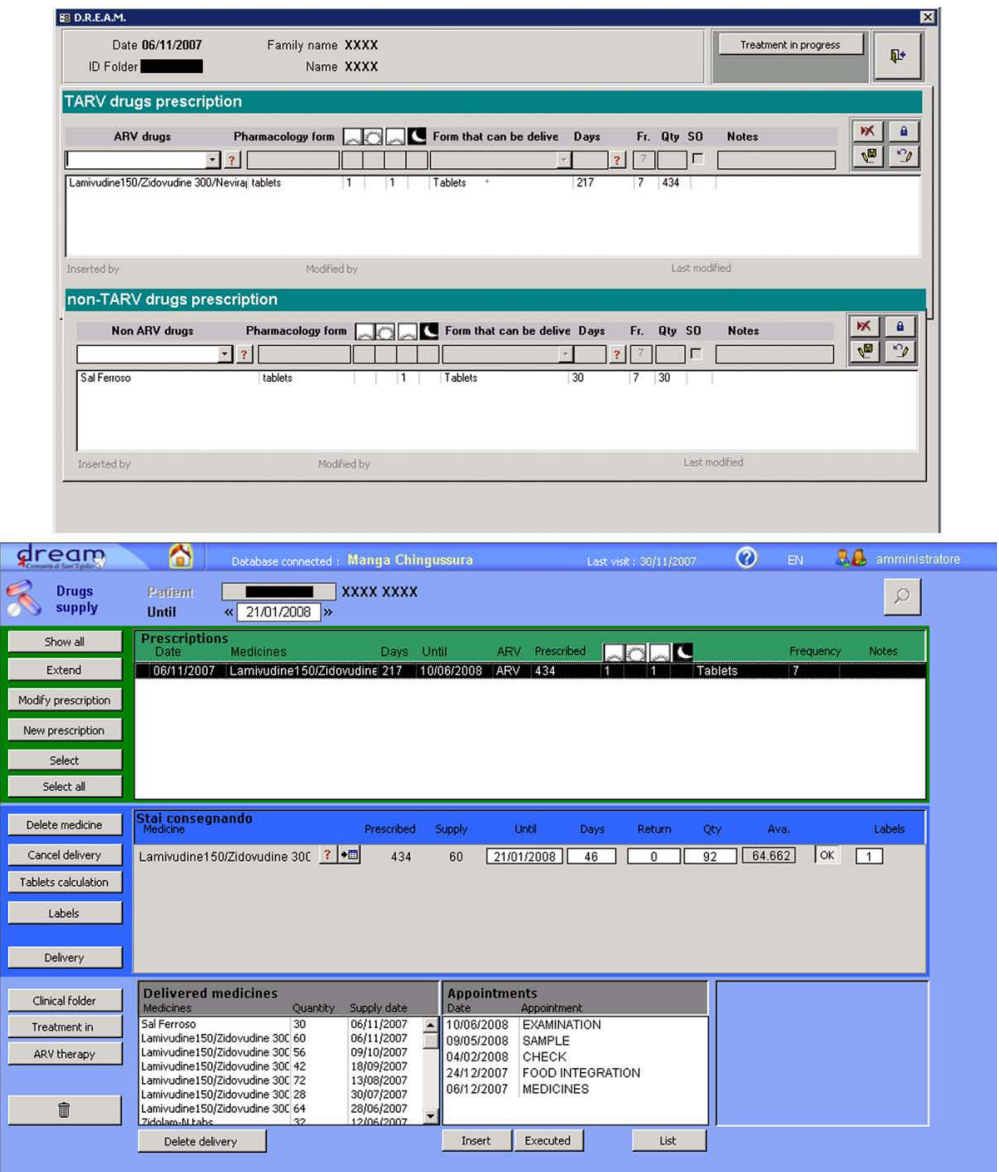
**A Screenshot of the DREAM Software's Drugs Management Form**.

Figure 5 shows two screenshots of the Drugs (prescriptions and handover) section of the software.

**Figure 6:**
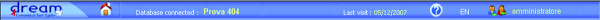
**A Screenshot of the DREAM Software's Status Bar**.

A very important function of the software is to be able to *change the language *used quickly, without having to reinstall the programme. In contexts like the one in which DREAM is active, where people of different nationalities (and languages) find themselves working together, this has turned out to be an essential instrument for overcoming errors and misunderstandings caused by the language used. Figure 6 shows how in the programme's status bar (at the top on the right) there is an option for the choice of language, which makes it possible to change from one language to another instantly without having to start the programme again, or even worse reinstall it.

## Results and discussion

In the years between 2002 and 2008, the DREAM programme grew considerably, in terms of both centres and patients on treatment. This expansion put the DREAM Software to the test and it has proved to be a effective tool, even when it comes to handling a large quantity of data.

The experience of DREAM Software is directly linked to the history of the DREAM programme. It was born of the needs met in the field, from the awareness that the complexity of treatment of people with HIV and AIDS calls for a high standard of quality to achieve success. On the other hand, DREAM is not a programme scheduled to come to a close.

The need for scalable solutions therefore emerged, not least from the point of view of the technologies used. Thus, we sought to identify all the possible IT solutions which could make the programme more efficient and effective, but which could at the same time be easily replicated and interoperable.

The expansion of DREAM in different countries has made the management of its software an important issue for a number of reasons, ranging from languages to different ways of thinking about the same problems. In fact today DREAM Software, which has reached its fourth version, is used in 31 centres throughout 10 countries in sub-Saharan Africa. A staff of around 20 technicians has been trained in specific courses for computer assistance in the DREAM centres. Moreover, the staff of developers in Europe guarantees a continuous tele-assistance service, which is accessible through the health centres' infrastructures (internet connection or telephone). Moreover, the need to integrate and share data has led to research for continuous improvements in communications, to make the sharing of data and experiences faster and more economical.

In any case we have sought to turn a need imposed by a contingency (that of having efficient management of clinical files and useful tools for the communication and sharing of data) into an opportunity. The personnel of the DREAM project have gained experience in the use of ICT, and have had the opportunity to raise problems and questions, which have proved useful for remodelling the software, providing the scope for redesigning the interfaces and proposing new functions that are useful in their daily work. In fact the DREAM staff take part in various refresher courses that are held several times a year. Since the beginning of the programme 14 training courses have been held, with 3,300 participants including doctors, nurses, biologists and social workers from over 10 African countries. During each of these courses there is a session on software training, whose aim is to extend the participants' knowledge of the software and evaluate the users' responses regarding its everyday use.

Furthermore, DREAM Software has proved to be a tool of crucial importance for epidemiological research. The fact that the treatment of people infected by HIV and AIDS is not yet widespread in sub-Saharan Africa poses problems for the management of therapies (the response to drugs, social factors, and so on), since the sickness is different from that found in Western countries, both due to the strain of the virus and to the socio-economic conditions involved. The data of over 73,000 medical files, 450,000 medical examinations and over 500,000 laboratory tests have been managed by the software up to today. The possibility of having such an extensive compilation of (standardised) data has therefore made it possible to refine strategies and guidelines for therapies [22-29], in such a way as to guarantee a continued evolution of the management of care and treatment, making it more and more effective. On the other hand, the fact that it has become possible to monitor each activity of the centres through the software (from examinations to tests, from the delivery of drugs to nutritional integration), allows for the assiduous and precise assessment of the quality of the services offered and the extent of resources employed. The management of drugs, for example, has constituted a critical aspect since the costs of medicines weigh heavily on the overall budget of the project.

Moreover, through the data collected, it is possible to give the national health services useful information for monitoring the epidemic. This is especially relevant when one bears in mind that DREAM is frequently a significant presence in the context of HIV/AIDS treatment, in those countries where it is at work. DREAM Software has tools that are capable of synthesising data collected and that have made it possible to streamline procedures for the production of reports. This feature is not a negligible one, because in projects like DREAM, a huge amount of resources are often employed for bureaucratic types of activity, such as providing national Governments or donors with updated reports about the situation of patients receiving care, the entity of resources used, or the general activities of the centres. Thus, streamlining these procedures means being able to dedicate more resources (human and economic) to care and treatment, and concentrate on the success of the programme.

## Conclusion

This paper illustrates the DREAM programme as well as one of its very important and sensitive aspects: the computer management of the patients' data. The attention paid to the requests of the end users, covering every aspect of care and treatment, the possibility of having homogenous data from different countries, are only some of the significant features which have made this computer system an indispensable tool for the management of treatment and for epidemiological research. All this has been achieved in places where minimal solutions, which are a far cry from Western standards, are often those proposed. In every DREAM treatment centre, a computer with the DREAM Software is available for all staff members; everyone follows the patient as per his/her specific competence and tasks and while doing so, has all the updated data of the said patient at his/her disposal. Not only does this make procedures more efficient and the work of the centre more streamlined, it has also served to guarantee the quality of data, with each staff member able to verify the said data from his/her own station.

Finally, the experience of DREAM overall, and of DREAM Software in particular, has shown how the catastrophe of HIV and AIDS in Africa also holds an opportunity: that of equipping the continent, in response to this emergency, with the necessary and appropriate technologies to overcome old and new problems: the digital divide, the poor possibilities of communications, the sprawling distances and poor logistics. The repercussions of the implementation of such technologies actually go well beyond the specific context of the epidemic. There are strong possibilities for the development of training too, since it would be possible, for example, to have long distance training with centres of excellence in Europe and elsewhere in the world. And this is a point of strategic priority [30].

## Competing interests

The authors declare that they have no competing interests.

## Authors' contributions

AN conceived of and designed the study and drafted the manuscript; GMB conducted interviews to gather the end users' expectations about the software, contributed to implement the software, and contributed to draft the manuscript; MB interpreted the data and revised the article; FDPM conducted interviews to gather the end users' expectations about the software and revised the article; PG interpreted the data, contributed to implement the software and revised the article; MP conducted interviews to gather the end users' expectations about the software, contributed to implement the software and contributed to draft the manuscript; GP interpreted the data and revised the article; LP conceived of and designed the study and revised the manuscript. All authors have read and approved the final manuscript.

## Pre-publication history

The pre-publication history for this paper can be accessed here:

http://www.biomedcentral.com/1472-6947/9/42/prepub
